# Genetic Structure and Preliminary Findings of Cryptic Diversity of the Malaysian Mahseer (*Tor tambroides* Valenciennes: Cyprinidae) Inferred from Mitochondrial DNA and Microsatellite Analyses

**DOI:** 10.1155/2013/170980

**Published:** 2013-12-26

**Authors:** Yuzine Esa, Khairul Adha Abdul Rahim

**Affiliations:** ^1^Department of Aquaculture, Faculty of Agriculture, Institute of Biosciences, Universiti Putra Malaysia, 43400 Serdang, Selangor, Malaysia; ^2^Department of Aquatic Science, Faculty of Resource Science and Technology, Universiti Malaysia Sarawak, 94300 Kota Samarahan, Sarawak, Malaysia

## Abstract

This study examines the population genetic structure of *Tor tambroides*, an important freshwater fish species in Malaysia, using fifteen polymorphic microsatellite loci and sequencing of 464 base pairs of the mitochondrial cytochrome c oxidase I (COI) gene. A total of 152 mahseer samples were collected from eight populations throughout the Malaysia river system. Microsatellites results found high levels of intrapopulation variations, but mitochondrial COI results found high levels of interpopulations differentiation. The possible reasons for their discrepancies might be the varying influence of genetic drift on each marker or the small sample sizes used in most of the populations. The Kelantan population showed very low levels of genetic variations using both mitochondrial and microsatellite analyses. Phylogenetic analysis of the COI gene found a unique haplotype (ER8∗), possibly representing a cryptic lineage of *T. douronensis*, from the Endau-Rompin population. Nevertheless, the inclusion of nuclear microsatellite analyses could not fully resolve the genetic identity of haplotype ER8∗ in the present study. Overall, the findings showed a serious need for more comprehensive and larger scale samplings, especially in remote river systems, in combination with molecular analyses using multiple markers, in order to discover more cryptic lineages or undescribed “genetic species” of mahseer.

## 1. Introduction

*Tor tambroides*, commonly known as mahseer, Kelah, or Empurau, is a highly priced cyprinid fish in Malaysia due to its delicious flesh and has a great potential for the freshwater aquaculture industry [1, 2]. It is also recognized as an excellent game fish and has a high demand in the ornamental fish industry due to its attractive color [2, 3]. Unfortunately, the natural habitat and effective population size of many freshwater fishes such as *T. tambroides* which require clean running water with gravels and rocks are rapidly being degraded due to anthropogenic disturbances (i.e., river pollution, deforestation, watershed erosion, and others) and uncontrolled fish harvesting [4]. Therefore, considering the economic importance of *T. tambroides* and given its fragmented distributions and population size, studies on the population structure and level of genetic variations throughout its distribution range are required for the effective management and conservation strategies of this important freshwater resource.

The phylogenetic relationship and taxonomic status between *T. tambroides* and its congeneric species *T. douronensis* have been conducted by several authors such as [5–7] who genetically confirmed their taxonomic status as distinct species. Esa et al. [8] subsequently examined the genetic structure of *T. tambroides* from 5 populations in Malaysia using the mitochondrial COI sequences and found low mitochondrial variations at the intrapopulations level but slightly higher variations at the interpopulation level. Although mitochondrial DNA has proven to be a powerful marker for detecting genetic subdivisions in many organisms [9], information provided by mtDNA alone is not always sufficient for genetics analysis due to its nature as a clonally and maternally inherited marker that carries genetic information only from female lineages [10].

In this study, we investigated the population structure of *T. tambroides* using microsatellites, a biparentally inherited nuclear marker. The highly variable microsatellites can provide a finer resolution of population-level dynamics, suitable for evolutionary and conservation genetics studies [13]. The microsatellites markers in the present study were used (a) to quantify the genetic diversity at the intra- and interpopulations levels, (b) to compare the observed genetic structure with those found by [8] using mitochondrial COI gene, and (c) to identify and discuss possible factors that could have influenced the population structure of *T. tambroides*.

## 2. Materials and Methods

### 2.1. Sample Collection and Identification

**Figure 1:**
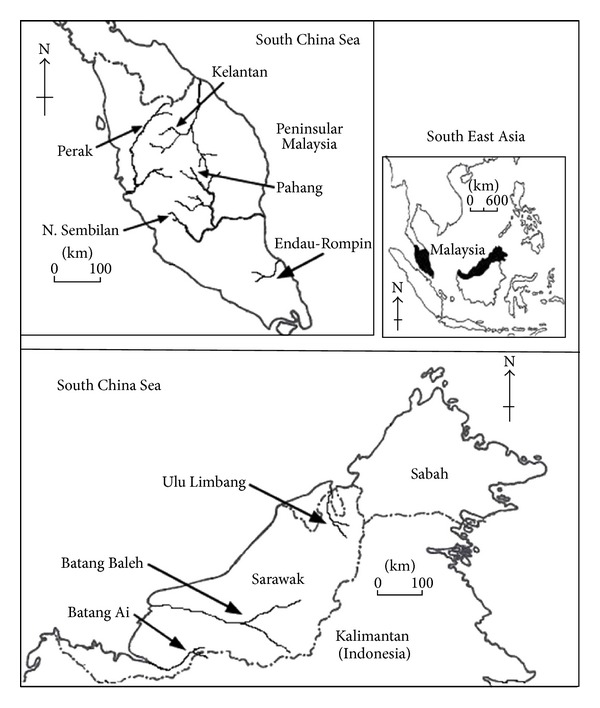
Map of sampling locations of *T. tambroides* in Malaysia.

Samples of *T. tambroides* used in this microsatellite study were similar to those described in [8] with the addition of 61 samples from the Endau-Rompin population located in the southern part of Peninsular Malaysia and five samples each from the Baleh and the Ulu Limbang populations of Sarawak, located in Borneo (Figure 1). All the *T. tambroides*, *T. douronensis,* and *Neolissochilus stracheyi* haplotypes found in [8] were also included with samples from three new locations mentioned above for the mitochondrial COI phylogenetic study. The fish samples (fin clipping, scale or whole fish) were preserved in 95% ethanol or kept on ice during field collection and were subsequently stored at −20°C prior to the genetic analyses. Morphological identification was done using keys provided by [14–16].

### 2.2. Mitochondrial DNA Polymerase Chain Reaction (PCR) and Sequencing

Total DNA was extracted using the CTAB method [17] in the presence of Proteinase K. The extracted genomic DNA was used for both mtDNA and microsatellite analyses. For mtDNA, a 500 bp segment of the *cytochrome c oxidase* I (COI) gene was amplified with the oligonucleotide primers COIf (5′ CCTGCAGGAGGAGGAGAYCC 3′, forward) and COIe (5′ CCAGAGATTAGAGGGAATCAGTG 3′, reverse) [18]. Approximately, 50–100 ng of the template DNA was amplified in a 25 *μ*L reaction mixture containing 50 mM 10X buffer, 2 mM MgCl_2_, 0.2 *μ*M of each dNTP (Promega), 0.1 *μ*M of each primer, and 0.5 units of *Taq* DNA polymerase (Promega). The cycle parameters consisted of 35 cycles of denaturation (95°C, 30 seconds), annealing (45°C, 30 seconds), and extension (72°C, 60 seconds). The amplified products were visualized on 2% agarose gel containing ethidium bromide, ran for approximately 30 min at 90 V, and photographed under UV light. The purified PCR products were directly sequenced using the BigDye Terminator v3.0 Cycle Sequencing kit (ACGT) on an ABI 377 automated sequencer (PE Applied Biosystem) using only the forward primer (COIf). A sequencing reaction using the reverse primer (COIe) was subsequently carried out on some of the samples (haplotypes) to verify the polymorphism in the DNA sequence initially detected using the forward primer.

### 2.3. Microsatellite Genotyping

Microsatellite analysis was carried out using 14 microsatellites loci as described in [19] and five polymorphic microsatellites loci found in a cross-species amplification study of *T. tambroides* using primers from other cyprinids [20]: *Cyprinus carpio* (MFW7 [21]), *Barbus barbus* (Barb37, Barb59, and Barb62 [22]), and *Barbonymus gonionotus* (Bgon13 [11]).

Polymerase chain reaction (PCR) amplifications were performed in a final volume of 10 *μ*L, containing 25–50 ng of genomic DNA, 1X PCR buffer (10 mM Tris-HCl, pH 9.0; 50 mM KCl; 0.01% gelatin), 2.0 mM MgCl_2_, 0.2 mM of each dNTP, 5 pmol of each primer, and 1.5 units of Taq DNA polymerase. Amplification conditions were 94°C for 5 min followed by 25 cycles at 94°C for 40 s, *T*_*a*_ (optimum annealing temperature for each primer pair) for 40 s and 72°C for 1 min, with a final extension of 72°C for 4 min.

Microsatellites were run on 4% high resolution MetaPhor agarose gels for 2 h at 78 V/cm, stained with ethidium bromide (0.1 *μ*L/mL), and photographed under UV light using an Alpha Imager 2200. The alleles were designated according to PCR product size and calculated relative to a standard molecular marker (20 bp and 100 bp; Cambrex and Promega).

### 2.4. Statistical Analyses

For mtDNA, multiple alignments of the COI sequences were conducted using the ClustalX program version 2.0.10 [23]. Test of saturation for all codons was done using DAMBE version 5.0.66 [24]. Phylogenetic relationships were inferred using four different methods of analysis: neighbor joining (NJ) [25], maximum parsimony (MP), maximum likelihood (ML), and Bayesian analysis. Modeltest 3.06 PPC [26] was used to identify the best model of evolution for the COI dataset. The model with the best maximum likelihood (ML) score using the Akaike Information Criterion (AIC) was chosen [27]. The best model suggested by the analysis was subsequently used in maximum likelihood (ML) and Bayesian analyses.

A distance analysis using the neighbor-joining method (NJ) was done using a close-neighbor-interchange (CNI) option implemented in MEGA (version 4.0 [28]). The NJ clustering was performed using the Kimura two-parameter evolution model [29]. Phylogenetic confidence was estimated by bootstrapping [30] with 1000 replicate data sets. The pairwise mean genetic distances between populations were also calculated using the Kimura two-parameter (K2P) model [29] implemented in MEGA.

A maximum parsimony (MP) tree was estimated using heuristic searches, as implemented in PAUP* v4.0b10 [12]. Heuristic searches were implemented using random addition sequence (100 repetitions) and tree bisection-reconnection (TBR) branch swapping procedure. Bootstrap trees [30] were computed using 1000 replicates.

Phylogenetic tree was also estimated using the maximum-likelihood (ML) approach also implemented in PAUP* v4.0b10 [12]. Bootstrap values were estimated using the same method as mentioned above but with 100 replicates and branch swapping. Bayesian analyses were performed using MrBayes version 3.0 [31]. The Markov Chain Monte Carlo (MCMC) process was set to 4 × 10^6^ generations with trees being sampled at every 100 generations.

For microsatellite analysis, the software package MICRO-CHECKER2.2.1 [32] was used to check for microsatellite genotyping errors due to null alleles, stuttering and large allele dropout for all loci and all populations. Microsatellite genetic diversity was characterized by estimates of allele richness (Ar), observed (*H*_*O*_), and expected (*H*_*E*_) heterozygosity [33] available in FSTAT version 2.9.3.2 [34]. Allele richness, rather than number of alleles, was computed to allow for a comparison among samples of different sizes [35]. GENEPOP version 3.4 [36] was used to test departures from the Hardy-Weinberg equilibrium (HWE) at each locus for each population based on the Markov chain method using 1000 dememorization steps, 100 batches, and 1000 iterations per batch. Genetic homogeneity of the data sets was determined through an exact test (*G*-based test) that assumes random samples of genotypes also using GENEPOP. Linkage disequilibrium to confirm independence between loci was tested by log-likelihood ratio *G*-tests (84,000 permutations) implemented in FSTAT. *F*-statistics for each locus and over all loci [37] and inbreeding coefficient (*F*_IS_) as a measure of heterozygote deficiency or excess [38] were calculated using FSTAT. Permutations (12,000 permutations) were used for testing the values of *F*_IS_ for significant departure from zero. The probability was estimated as the proportion of permutations which gave as large or larger statistic as the observed one. Sequential Bonferroni adjustments were made for all multiple comparisons [39].

The significance of the spatial variation in gene diversity of *T. tambroides* populations was estimated by performing a hierarchical analysis of genetic diversity using the analysis of molecular variance (AMOVA) using ARLEQUIN version 3.01 [40]. Differentiation among populations was measured by the fixation index *F*_ST_, calculated according to [37], also using ARLEQUIN. Permutation tests (10,000 permutations) were performed in order to determine if estimates differed significantly from zero. The genetic distance between populations (rivers) in both mahseers was calculated based on an unbiased measure following [41]. An Unweighted Pair-Group Method with Arithmetic Mean (UPGMA) dendrogram was constructed to illustrate the relations among geographic samples using POPGENE 1.32 [42].

To compute the probability that an individual belonged to each reference population, assignment tests were performed on the basis of multilocus genotype data using GENECLASS version 2.0 [43]. The Bayesian method of [44] was used as the computation criterion and resampling algorithm based on [45] was employed. Data was running using 10,000 simulations and a threshold of significance *α* = 0.01.

A Bayesian approach was used to infer the number of clusters (*K*) in the data set without prior information of the sampling locations, available in STRUCTURE version 2.0 [46]. A model where the allele frequencies were correlated within populations was assumed (*λ* was set at 1, the default value). The software was run with the option of admixture, allowing for some mixed ancestry within individuals, and *α* was allowed to vary. Ten independent runs were done for each value of *K* (*K* = 1 to 8 for each species) with a burn-in period of 25,000 iterations and 25,000 replications.

Finally, evidence of a recent bottleneck for each population was tested using BOTTLENECK 1.1 [47]. BOTTLENECK tests for a significant heterozygosity excess were compared to equilibrium expectations for a stable population based on the assumption that population reductions cause rare alleles to be lost faster than genetic diversity, resulting in a transient heterozygosity excess compared to the observed number of alleles [48]. The two-phase mutation (TPM) model, which is considered appropriate for microsatellites [49], was applied with a variance of 12 [47] and different percentages of the stepwise mutation model (SMM: 70, 80, and 90%). In all bottleneck analyses, 1,000 iterations were used, with results over loci being derived from the Wilcoxon signed-rank test recommended by [50]. To complement the mutational model-based analyses, we also collated evidence for the presence of skewed distributions of allele frequencies within each population [51]. Although this approach does not constitute a formal statistical test, detection of these pattern types can also provide heuristic evidence for a recent bottleneck [51].

## 3. Results

### 3.1. Mitochondrial COI Characterization

For the whole COI dataset, there were 87 (18.8%) variable/polymorphic sites including 56 (12.1%) parsimony-informative sites while 377 (81.2%) were conserved sites. The mean total of the nucleotide composition was 25.7% A, 32.3% T, 22.6% C, and 19.4% G. In total, 24 substitutions were found among haplotypes, of which there were 21 transitions and three transversions. All substitutions occurred at the third codon position, a common characteristic of mitochondrial DNA [52]. The protein translation of the 464 bp fragment retained only 14 haplotypes in the amino acid sequence, comprising 154 amino acid residues, of which 15 (9.7%) were variable sites. The translation result indicated no sign of pseudogene in the amino acid sequences while saturation analysis showed no signs of saturation at the third codon position.

**Figure 2:**
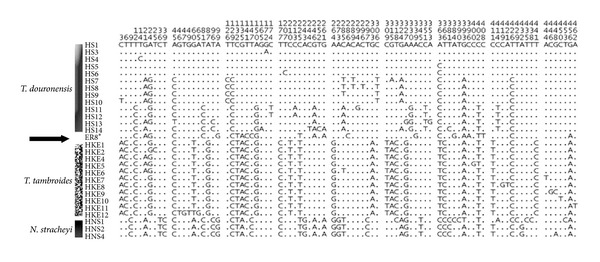
Summary of variable sites of 28 observed mitochondrial DNA cytochrome c oxidase I (COI) haplotypes of the mahseer including haplotype ER8*. Dots indicate identity with the HS1 haplotype sequence. HS: *T. douronensis* haplotypes; HKE: *T. tambroides* haplotypes; HNS: *N. stracheyi* haplotypes.

**Figure 3:**
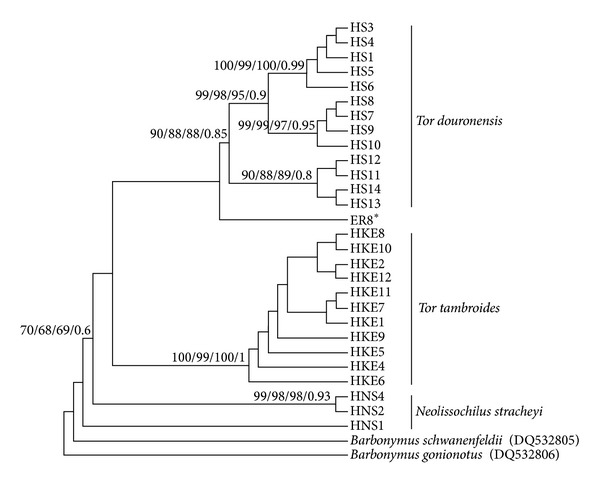
Phylogenetic analyses tree showing relationships among the mahseer haplotypes and haplotype ER8* using 4 different methods: neighbor joining (NJ), maximum parsimony (MP), maximum likelihood (ML), and Bayesian analysis. The number at each node represents the bootstrap value (%) or posterior probabilities (Bayesian) of the dataset.

Phylogenetic analyses using all methods (NJ, MP, ML, and Bayesian) generally produced similar tree topologies with slight differences in their bootstrap support values; thus only the Bayesian tree was chosen to show the mahseer phylogenetic relationships. Accordingly, the COI sequences of the 71 samples of mahseers from the Endau-Rompin, Baleh and Ulu Limbang populations produced a total of only 4 haplotypes with two unique haplotypes (HKE12 and ER8*) were only found from the Endau-Rompin population (Figure 2). All sequences from the Ulu Limbang population clustered together into haplotype HKE6 while all sequences from the Baleh population clustered into haplotype HKE4, the most common haplotype found from the Borneo population. For the Endau-Rompin population, the majority of the sequences (*n* = 47) clustered into haplotype HKE4, while haplotype HKE12 comprising a single sequence was clustered within the *T. tambroides* group. Interestingly, ER8* which was another unique haplotype that consisted of four sequences was positioned intermediately between the *T. tambroides* and *T. douronensis* clusters based on all methods of phylogenetic analyses (Figure 3). Thus, the genetic identity of the cryptic haplotype ER8* could not be confirmed based on COI sequence analyses.

### 3.2. Microsatellites Polymorphism

MICRO-CHECKER revealed evidence of a general excess of homozygotes for most allele size classes at three loci (Tt1.B01, Barb37, and Barb62). There were no indications of scoring errors attributable to stuttering or large allele dropout. The general excess of homozygotes for most allele size classes may hence indicate the presence of null alleles or a Wahlund effect. For the microsatellites developed by [19], ten out of 13 loci were polymorphic in one or more populations. Thus, together with the additional five polymorphic loci, this resulted in a total of 15 polymorphic loci found in *T. tambroides* (Table 1). Polymorphism at microsatellite loci varied with allelic richness per locus ranging between 1.2110 (Bgon13) and 4.6670 (Tt1.B02). Expected heterozygosity within each population ranged between 0.0163 (MFW7 of Endau-Rompin) and 0.7900 (Tt1.B02 of Negeri Sembilan).

### 3.3. Hardy-Weinberg Equilibrium and Linkage Disequilibrium

Randomization tests showed that six (40.0%) of the 15 single locus permutation tests were inconsistent with Hardy-Weinberg equilibrium expectations in *T. tambroides*. However, only two loci (Tt2.B02 and Barb62) still displayed significant deviations from Hardy-Weinberg expectations after adjustments with Bonferroni correction. In all populations, *F*_IS_ values were significantly different from zero (*P* < 0.05) in both species except in the Endau-Rompin population, indicating a loss of heterozygosity within this population in accordance with significant deviations from Hardy-Weinberg equilibrium. Linkage disequilibrium among pairs of loci was tested for 105 combinations over 15 loci with 26 (20.5%) that were in linkage disequilibrium (only one significant deviation after Bonferroni correction). After pooling all populations, 12 comparisons were highly significant (only one after Bonferroni correction).

### 3.4. Genetic Differentiation among Populations and Species

AMOVA results revealed that the majority of variance (83.94%) was from within individual variations, and only 13.13% of total variance resulted from inter-population difference (Table 2). Pairwise estimates of *F*_ST_ over all loci showed that 22 (78.5%) out of 28 pairwise estimates showed significant genetic differentiation with the Kelantan population showing the highest degree of differentiation from all other populations (*F*_ST_ = 0.1811–0.6494, *P* < 0.05) (Table 3). In all populations, *F*_ST_ values were significantly different from zero (*P* < 0.01). Pairwise estimates of genetic distances computed by [33] among populations are shown in Table 3. The highest genetic distance was between the Kelantan population and the Perak population (0.1709).

Results of the assignment tests using GENECLASS2 showed that on average 42.8% (66 out of 152) of the individuals were correctly assigned to their original sampling site (Table 4). The Perak population presented the highest percentage of correctly assigned individuals (72.6%). However, none of the individuals were correctly assigned to their original sampling locations in the Kelantan (all 20 individuals were misassigned to the Endau-Rompin population), the Baleh (three individuals were misassigned to the Pahang and two individuals to the Endau-Rompin populations), and the Ulu Limbang (three individuals were misassigned to the Pahang and one individual to the Baleh and the Batang Ai populations each) populations of Sarawak.

**Figure 4:**
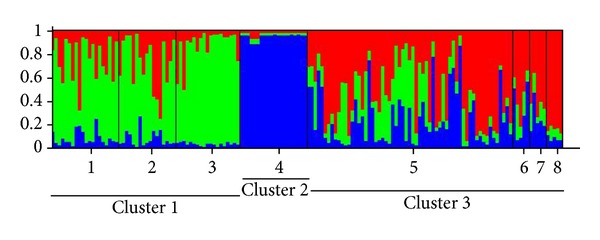
Proportional membership (Q) of each individual of *T. tambroides* in the three clusters identified by STRUCTURE. The numbers on the *x*-axis correspond to a specific population: 1: Negeri Sembilan, 2: Pahang, 3: Perak, 4: Kelantan, 5: Endau-Rompin, 6: Baleh, 7: Ulu Limbang, and 8: Batang Ai.

Bayesian cluster analysis performed with STRUCTURE showed that the most likely *K* value identified for *T. tambroides *was *K* = 3 (Figure 4). The three clusters are (i) Cluster I: Negeri Sembilan, Pahang, and Perak, (ii) Cluster II: Kelantan, and (iii) Cluster III: Batang Ai, Baleh, and Ulu Limbang of Sarawak. The UPGMA dendrogram also produced similar clusterings within the *T. tambroides* populations (Figure 6) as identified by STRUCTURE.

In bottleneck analyses, we found supporting evidence for recent reductions in population size within the Perak, Endau-Rompin, Batang Ai, and Ulu Limbang populations using the two-phase model (Table 5). In addition, evidence from the infinite allele model suggested bottlenecks within the Perak population while the stepwise mutation model supported that bottlenecks are also present within the Endau-Rompin, Batang Ai, and Ulu Limbang populations. Nevertheless, a mode shift in the allele-frequency spectrum was detected in four populations including the Negeri Sembilan population, although no significant result was found in the bottleneck analyses using all mutational models in that particular population. No evidence of a recent bottleneck was found within the Kelantan population (Table 5).

## 4. Discussion

### 4.1. Genetic Diversity and Population Differentiation

The mean expected heterozygosity (0.3184) at 15 microsatellite DNA loci found in *T. tambroides *was lower than the mean heterozygosity (0.5400) reported in most freshwater fish species (13 species [53]) but was comparable with those found in other studies of mahseer population structure using microsatellites [20, 54–56].

Empirical studies of natural populations have commonly reported departures from Hardy-Weinberg equilibrium in a wide range of fish species [57–59]. Observed heterozygote deficits could be caused by a number of factors including, inbreeding, intra-population structure (Wahlund effect) [58], a founder event, existence of null alleles, selection against heterozygotes [60], fishing pressure [61], or combinations of these above-mentioned factors. Nonrandom sampling methods (sampling bias) would also be a possible reason (e.g., sample size of the Batang Ai, Baleh, and Ulu Limbang populations were small (<10 individuals) so that one of the alleles could not be detected), but sample sizes of other populations were sufficiently large enough (Table 1).

The results of the microsatellite analyses showed mixed levels of population differentiation among extant populations of *T. tambroides *(*F*_ST_: 0.0011 to 0.6494, genetic distances: 0.2% to 17.1%). Nevertheless, significant differentiation in 78.5% of the pairwise comparisons among populations from the eight rivers confirmed their population genetic divergence. AMOVA analyses further supported the low inter-population differentiation among the *T. tambroides* populations with the majority of variation resulting from inside the populations compared to only 13% among populations. The pattern of genetic structure of the *T. tambroides* populations based on the present microsatellite analyses is most likely to be modulated by historically high gene flow between populations and then followed by genetic isolation. The mixed pattern of population differentiation was further supported by the assignment test results where samples from the Perak population showed a high percentage of correctly assigned individuals, followed by a moderate percentage in the Pahang and the Endau-Rompin populations, and a low percentage in other populations. Accordingly, a high percentage of correctly assigned individuals may indicate strong genetic divergence among populations and, conversely, low assignment success may indicate weak divergence [45].

### 4.2. Comparisons of Microsatellite Data with Mitochondrial Sequences Data

The patterns of genetic variations differed between results generated using microsatellites in the present study and results from mitochondrial COI sequences done by [8] in *T. tambroides*. Microsatellites results found high levels of within- (intra)population variations in the present study, but mitochondrial results found high levels of among- (inter)populations differentiation. Furthermore, microsatellites results showed low genetic differentiation among the three populations of Peninsular Malaysia (Negeri Sembilan, Pahang, and Perak), but significant differentiation was found between them and the Kelantan population, which was slightly different compared to the overall low genetic differences found among the four populations observed using a mitochondrial dataset [8]. The population structuring among the other *T. tambroides* populations was evidently higher based on comparing mitochondrial and microsatellite dataset.

Thus, the discrepancies between the mitochondrial and microsatellite datasets regarding the *T. tambroides* population structure can be discussed based on two assumptions. Firstly, differences between these datasets may reflect the varying influence of genetic drift on mitochondrial and nuclear genomes. Mitochondrial genes have a 4-fold lower effective population size, relative to their diploid/biparentally inherited nuclear counterparts, due to their uniparental (maternal) inheritance and haploid genotypes [62]. Given this reduced effective population size, mitochondrial alleles are much more susceptible to processes such as genetic drift and are therefore more likely to demonstrate strong patterns of genetic structure relative to nuclear alleles over comparable evolutionary time scales. Secondly, the apparent lack of microsatellites variations among the *T. tambroides* populations was due to the small sample sizes in most of the populations, where some of the alleles could not be detected, thus reducing their overall level of genetic variations [63].

The microsatellite results also showed very low genetic variation in the *T. tambroides* population from Kelantan (only one polymorphic loci = Barb59 from a single fish sample out of a total of 15 loci analyzed), which is consistent with a low mitochondrial variation (a single haplotype = HKE1 in all samples) found by [8]. Thus, the lack of genetic variations in the Kelantan population could be due to null alleles or other biological phenomena such as habitat destruction or overexploitation. In the present study, heterozygotes deficits may result from the occurrence of null alleles, as observed at three microsatellite loci (Tt1.B01, Barb37, and Barb62) in *T. tambroides*. Null alleles are alleles that are not amplified during PCR because of mutation events changing the DNA sequence in one of the primer sites (mostly at the 3′ end), which causes the primer to no longer anneal to the template DNA during PCR [32]. Subsequently, this may prevent certain alleles from being efficiently amplified by PCR. This results either in no PCR product, if a null allele is homozygote, or in false homozygote individuals, if the locus is a heterozygote. This will show apparent significant deviations from Hardy-Weinberg equilibrium and non-Mendelian inheritance of alleles [64]. Nevertheless, the extremely low levels of mitochondrial COI variation found within the Kelantan population (fixed to a single haplotype) make the null allele factor of the microsatellite loci highly unlikely. In addition, there was no instance of nonamplifying samples in repeated trials in any of the primers in *T. tambroides*. Hence, in the present study, the possible causes for an excess of homozygosity can be speculated as habitat destruction and overexploitation in the upper reaches/headwaters of the Kelantan River, especially the northwestern part of the headwater, where the samples might have been collected. This is because mass reduction to the effective population size of *T. tambroides* in this area would therefore lead to large reductions in both mitochondrial and microsatellite variations. Although bottleneck analyses showed no evidence of a recent bottleneck occurring within the Kelantan population (Table 5), the power of the mode shift allele-frequency spectrum analyses to detect recent bottleneck is highly dependent on the number of individuals sampled [51]. The ability to detect previous population bottleneck is also influenced by the magnitude of the past reduction in population size [51] with larger population reductions following the original disturbance being more easily detected.

Unfortunately, the identity of the Kelantan samples could not be fully verified because the samples were supplied to us (in the form of muscle tissues and fin clips) without any information regarding their specific location of capture and detailed morphological description by a private conservation company. Therefore, we suggest that a fine scale genetic analysis using multiple markers must be done throughout the upper reaches of Kelantan River in order to confirm the actual level of genetic variation in the *T. tambroides* population of Kelantan.

### 4.3. Genetic Identity of Haplotype ER8*

Phylogenetic analysis found a unique haplotype lineage (ER8*) from four fish individuals from the Endau-Rompin population. This particular haplotype showed conflicting phylogenetic positioning within both the *T. douronensis *and the *T. tambroides *clades, instead of a grouping with the other Endau-Rompin haplotypes in the *T. tambroides* clade (Figure 3). The pairwise genetic distances showed that the haplotype ER8* was genetically closer to *T. douronensis *(4.5%–5.9%) compared to *T. tambroides* (5.9%–6.6%). This finding was of particular interest because the *T. douronensis* lineage has never been described from rivers in Peninsular Malaysia, although their distributions commonly overlap with *T. tambroides* in the upper streams rivers of the Sarawak state of the Borneo Island [1].

Thus, analysis of hypervariable microsatellite loci was conducted in order to provide further information regarding the genetic identity of haplotype ER8*. The *T. douronensis* samples from a few populations (Batang Ai, Layar, Bario, Ba Kelalan, Ulu Limbang, and Sabah) as well as the four individuals representing the ER8* haplotype (grouped together as a single population) were included in the analyses. Microsatellite analysis showed marked differences in allelic frequencies at a few loci between *T. tambroides* and *T. douronensis *with both species harbouring private alleles, although no fully diagnostic loci could be found between them (data not shown). Accordingly, *T. douronensis* samples exhibited a low “120” private allele at the Tt2.D01 loci not found in the *T. tambroides* populations while the *T. douronensis* samples also lacked allele “180” at the Tt1.C10 loci which was found at high frequencies in the *T. tambroides* populations. In addition, all the *T. douronensis *populations also exhibited high frequencies of allele “140” to allele “160” at the Barb62 loci compared with a lower allelic range (allele “108” to allele “120”) commonly found in the *T. tambroides* populations.

**Figure 5:**
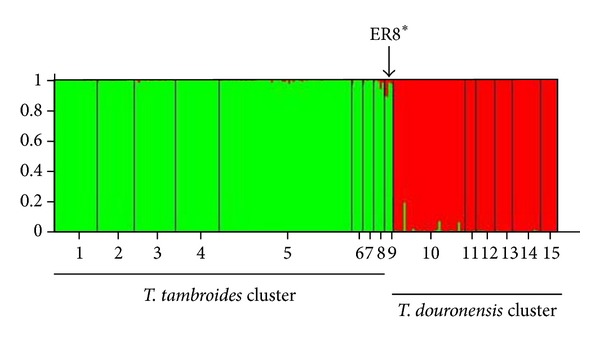
Proportional membership (Q) of each individual of *T. tambroides*, *T. douronensis,* and ER8* in the clusters identified by STRUCTURE. The numbers on the *x*-axis correspond to a specific population: 1 to 8: *T. tambroides* populations, 10 to 15: *T. douronensis* populations, and 9: ER8*.

**Figure 6:**
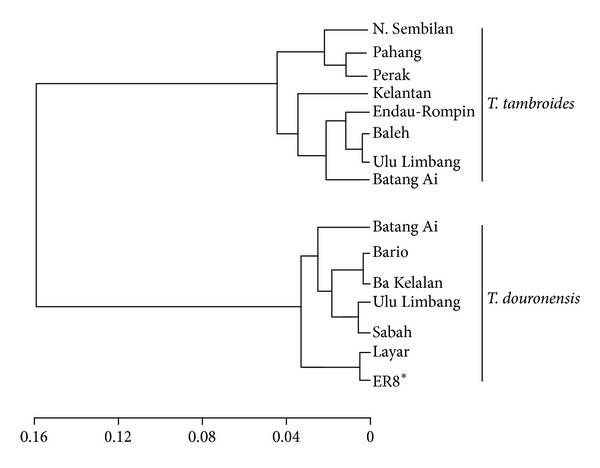
UPGMA cluster diagram based on [11] unbiased genetic distance for eight populations of *T. tambroides*, six populations of *T. douronensis,* and mahseer samples of haplotype ER8* based on 13 microsatellite loci.

Interestingly, all the four putative mahseer individuals generally possessed allelic frequencies characteristics of both *T. tambroides* and *T. douronensis *at different frequencies but also exhibited a few private alleles themselves (allele “240” at the Tt1.A06 loci, allele “230” at the Tt1.C10 loci, and allele “152” at the Tt2.B02 loci). Results from Bayesian cluster analysis (Figure 5) clustered the haplotype ER8* samples together with the other *T. tambroides *samples with a high proportional membership of 92.8%. However, the UPGMA dendrogram provided a different result where samples of haplotype ER8* were grouped together within the *T. douronensis* cluster although their relationship was weakly supported since a tied tree was found in the cluster analysis (Figure 6). Thus, the inclusion of nuclear microsatellite datasets in this study still could not fully resolve the genetic identity of haplotype ER8*.

Therefore, several possibilities could be made regarding the genetic identity of haplotype ER8* based on both the microsatellite and mitochondrial results of the current study. First, haplotype ER8* could represent a cryptic *T. douronensis* lineage found for the first time in Peninsular Malaysia. This was supported by (i) the clustering of haplotype ER8* within the *T. douronensis* clade based on phylogenetic analyses of COI sequence as well as UPGMA analysis of microsatellite data sets and (ii) its closer genetic relationship with other *T. douronensis* haplotypes compared with *T. tambroides* haplotypes.

Furthermore, the possible occurrence of a *T. douronensis* lineage in the Endau-Rompin River can be explained in relation to the biogeographical history of South East Asia (historically known as the Sundaland). Geological evidence suggests that the river systems of the southern parts of Sarawak were historically interconnected with most major river systems of Peninsular Malaysia during the Tertiary and Quaternary periods (10–5 Ma) via the North Sunda River [65], thus allowing gene flow among these drainages [8]. The rise of sea levels during the last Pleistocene period eventually separated Borneo from mainland Asia (Peninsular Malaysia) which ultimately resulted in the isolation of *T. douronensis* of the Endau-Rompin populations from their Borneo population counterparts. Similar evidence of a close genetic relationship between freshwater fishes of Borneo and mainland Asia in relation to their biogeographical history was discussed by several other authors including [9, 66] in relation to *Hemibagrus nemurus* and *Hampala macrolepidota*, respectively.

Secondly, haplotype ER8* might represent a cryptic lineage of *T. tambroides* itself in the Endau-Rompin population but with a high genetic divergence compared with the other *T. tambroides* haplotypes which exhibited slight differences in their allelic frequencies as well. The high proportional membership of microsatellites data into the *T. tambroides* cluster (92.8%) identified by STRUCTURE further supported this hypothesis. Thus, the higher number of samples analyzed from the Endau-Rompin population has also increased the chances of detecting new or cryptic lineage compared with smaller samples sizes analyzed from other populations.

Thirdly, ER8* presumably represent a hybrid population between *T. tambroides *and *T. douronensis* since freshwater fishes, such as cyprinids, commonly hybridize in nature [67]. The sharing of some allelic frequencies characteristic of both *T. tambroides* and *T. douronensis* could indicate that they were hybrids, possibly from backcross generations. However, we lack a fully diagnostic microsatellite loci and, along with an unresolved phylogenetic relationship of ER8* haplotype, restricted us from confirming this hybridization issue. On the other hand, it is worthy to note that no hybrid individuals were found based on our genetic analysis from the other sympatric locations of the two mahseer in Sarawak such as in the Batang Ai and the Ulu Limbang populations. This appears to suggest that a hybridization event between *T. tambroides* and *T. douronensis* might be absent (but is not impossible) due to undetermined reproductive isolation factors. Nevertheless, further studies are needed using more microsatellite loci and larger sample sizes from their sympatric locations in order to reconcile and confirm the hybridization issue.

In summary, this study has managed to provide further insights into the population structure and levels of genetic diversity among *T. tambroides* populations in Malaysia. The utilization of both the mitochondrial and microsatellites markers showed different degrees of genetic differentiation and levels of resolution within and among populations, but their combination proved to be a better choice for future population genetic studies of mahseer as well as other freshwater species. The preliminary findings of a potential cryptic lineage of *T. douronensis* from the Endau-Rompin population showed a need for more comprehensive and larger scale surveys, especially in remote river systems in order to find more cryptic lineages or undescribed “genetic species” of mahseer.

## Figures and Tables

**Table 1:** Genetic variability at 15 microsatellite loci from eight populations in *T. tambroides*.

Locus	N. Sembilan	Pahang	Perak	Kelantan	Endau-Rompin	Baleh	Ulu Limbang	Batang Ai
Tt1.A06								
*N*	20	17	19	20	61	5	5	5
Ar	1.0000	1.0000	1.0000	1.0000	1.3530	2.0000	2.0000	2.0000
*H*_*O*_	0.0000	0.0000	0.0000	0.0000	0.0820	0.2000	0.2000	0.2000
*H*_*E*_	0.0000	0.0000	0.0000	0.0000	0.0786	0.1800	0.1800	0.1800
*F*_IS_	—	—	—	—	−0.0340	0.0000	0.0000	0.0000
HW	—	—	—	—	1.0000	1.0000	1.0000	1.0000
Tt1.B01								
*N*	20	17	19	20	61	5	5	5
Ar	1.7000	1.2940	1.7230	1.0000	1.4080	2.0000	2.0000	2.0000
*H*_*O*_	0.0000	0.0588	0.1053	0.0000	0.0984	0.4000	0.2000	0.2000
*H*_*E*_	0.1800	0.0571	0.1884	0.0000	0.0935	0.3200	0.1800	0.1800
*F*_IS_	1.0000	0.0000	0.4630	—	−0.0430	−0.1430	0.0000	0.0000
HW	0.0019*	1.0000	0.1656	—	1.0000	1.0000	1.0000	1.0000
Tt1.B08								
*N*	20	17	19	20	61	5	5	5
Ar	1.9500	2.1410	2.2520	1.0000	1.8170	2.0000	1.0000	2.0000
*H*_*O*_	0.2500	0.3529	0.6842	0.0000	0.2131	0.2000	0.0000	0.2000
*H*_*E*_	0.2238	0.2993	0.4668	0.0000	0.1944	0.1800	0.0000	0.1800
*F*_IS_	−0.0920	−0.1500	−0.4444	—	−0.0880	0.0000	0.0000	0.0000
HW	1.0000	1.0000	1.0000	—	1.0000	1.0000	—	1.0000
Tt1.C06								
*N*	20	17	19	20	61	5	5	5
Ar	1.9760	1.7710	1.9890	1.0000	1.8110	2.0000	2.0000	2.0000
*H*_*O*_	0.4500	0.2353	0.5263	0.0000	0.1311	0.4000	0.2000	0.2000
*H*_*E*_	0.3987	0.2076	0.4321	0.0000	0.2515	0.3200	0.1800	0.1800
*F*_IS_	−0.1030	−0.1030	−0.1920	—	0.4850	−0.1430	0.0000	0.0000
HW	0.8523	1.0000	0.9207	—	0.0019*	1.0000	1.0000	1.0000
Tt1.C10								
*N*	20	17	19	20	61	5	5	5
Ar	2.7480	2.6530	2.7210	1.0000	2.1190	2.0000	2.0000	3.0000
*H*_*O*_	0.5500	0.7059	0.9474	0.0000	0.2951	0.2000	0.2000	0.6000
*H*_*E*_	0.5312	0.5415	0.5873	0.0000	0.2743	0.1800	0.1800	0.4600
*F*_IS_	−0.0100	−0.2760	−0.5960	—	−0.0680	0.0000	0.0000	−0.2000
HW	0.6178	0.9580	1.0000	—	0.8103	1.0000	1.0000	1.0000
Tt2.B02								
*N*	20	17	19	20	61	5	5	5
Ar	4.6670	3.9050	4.0710	1.0000	3.2550	3.0000	3.0000	2.0000
*H*_*O*_	0.9500	0.8824	0.8421	0.0000	0.3770	0.8000	0.8000	0.6000
*H*_*E*_	0.7900	0.7111	0.7271	0.0000	0.6340	0.6400	0.5600	0.4200
*F*_IS_	−0.1780	−0.2120	−0.1320	—	0.4120	−0.1430	−0.3330	−0.3330
HW	0.9865	0.9719	0.9084	—	0.0001*	0.7943	1.0000	1.0000
Tt2.B10								
*N*	20	17	19	20	61	5	5	5
Ar	3.6560	3.7210	3.7820	1.0000	3.0810	2.0000	2.0000	2.0000
*H*_*O*_	0.7500	0.5294	0.8421	0.0000	0.7213	0.4000	0.6000	0.4000
*H*_*E*_	0.7037	0.7076	0.6994	0.0000	0.6070	0.3200	0.4200	0.3200
*F*_IS_	−0.0400	0.2800	−0.1780	—	−0.1800	−0.1430	−0.3330	−0.1430
HW	0.7113	0.0516	0.9462	—	0.9815	1.0000	1.0000	1.0000
Tt2.D01								
*N*	20	17	19	20	61	5	5	5
Ar	3.6320	3.3930	3.6980	1.0000	3.2120	4.0000	3.0000	4.0000
*H*_*O*_	0.8000	0.7059	0.5263	0.0000	0.5082	0.8000	0.8000	0.6000
*H*_*E*_	0.7112	0.6574	0.7036	0.0000	0.5849	0.5800	0.6200	0.4800
*F*_IS_	−0.099	−0.0430	0.2770	—	0.1390	−0.280	−0.1850	−0.143
HW	0.8410	0.7013	0.0476	—	0.0721	1.0000	0.8703	1.0000
Tt2.F04								
*N*	20	17	19	20	61	5	5	5
Ar	1.9990	1.9990	2.0000	1.0000	1.8610	2.0000	2.0000	2.0000
*H*_*O*_	0.2500	0.2941	0.2105	0.0000	0.3115	0.2000	0.2000	0.4000
*H*_*E*_	0.4988	0.4931	0.4986	0.0000	0.2850	0.1800	0.1800	0.3200
*F*_IS_	0.5180	0.4290	0.5960	—	−0.0850	0.0000	0.0000	−0.1430
HW	0.0259*	0.0932	0.0127*	—	0.8735	1.0000	1.0000	1.0000
Tt2.H08								
*N*	20	17	19	20	61	5	5	5
Ar	1.9760	2.0000	1.9930	1.0000	1.6600	2.0000	2.0000	2.0000
*H*_*O*_	0.5500	0.6471	0.5789	0.0000	0.1967	0.6000	0.2000	0.4000
*H*_*E*_	0.3987	0.5000	0.4501	0.0000	0.1774	0.4200	0.1800	0.3200
*F*_IS_	−0.3570	−0.2660	−0.2610	—	−0.1010	−0.3330	0.0000	−0.1430
HW	1.0000	0.9478	0.9615	—	1.0000	1.0000	1.0000	1.0000
MFW7								
*N*	20	17	19	20	61	5	5	5
Ar	1.0000	1.9000	1.8640	1.0000	1.0820	1.0000	2.0000	1.0000
*H*_*O*_	0.0000	0.3529	0.3158	0.0000	0.0164	0.0000	0.4000	0.0000
*H*_*E*_	0.0000	0.2907	0.2659	0.0000	0.0163	0.0000	0.3200	0.0000
*F*_IS_	—	−0.1850	−0.1610	—	0.0000	—	−0.1430	—
HW	—	1.0000	1.0000	—	1.0000	—	1.0000	—
Barb37								
*N*	20	17	19	20	61	5	5	5
Ar	2.2400	2.6210	2.7100	1.0000	1.2930	2.0000	2.0000	2.0000
*H*_*O*_	0.5500	0.2353	0.2632	0.0000	0.0328	0.2000	0.2000	0.2000
*H*_*E*_	0.4712	0.4792	0.4806	0.0000	0.0634	0.1800	0.3200	0.1800
*F*_IS_	−0.14200	0.5310	0.4740	—	0.4890	0.0000	0.0000	0.0000
HW	0.7888	0.0076*	0.0093*	—	0.0548*	1.0000	1.0000	1.0000
Barb59								
*N*	20	17	19	20	61	5	5	5
Ar	2.8350	3.9350	4.4650	1.5890	4.3630	2.0000	2.0000	3.0000
*H*_*O*_	0.5500	1.0000	0.8421	0.1500	0.7213	0.2000	0.2000	0.8000
*H*_*E*_	0.4712	0.6816	0.7604	0.1387	0.7309	0.4200	0.1800	0.5400
*F*_IS_	0.1590	−0.4410	−0.0810	−0.0560	0.0210	0.6000	0.0000	−0.3910
HW	0.2528	1.0000	0.8334	1.0000	0.4343	0.3298	1.0000	1.0000
Barb62								
*N*	20	17	19	20	61	5	5	5
Ar	2.3450	2.7070	2.4350	1.0000	3.5770	2.0000	3.0000	4.0000
*H*_*O*_	0.1500	0.4118	0.5263	0.0000	0.3934	0.2000	0.4000	0.8000
*H*_*E*_	0.3362	0.4682	0.4598	0.0000	0.6088	0.4200	0.3400	0.7000
*F*_IS_	0.5710	0.1820	−0.1180	—	0.3610	0.6000	0.0000	−0.3910
HW	0.0046*	0.2479	0.8263	—	0.0001*	0.3366	1.0000	0.7168
Bgon13								
*N*	20	17	19	20	61	5	5	5
Ar	1.4420	1.5080	1.6120	1.0000	1.0000	1.0000	1.0000	1.0000
*H*_*O*_	0.1000	0.1176	0.1579	0.0000	0.0000	0.0000	0.0000	0.0000
*H*_*E*_	0.0950	0.1107	0.1454	0.0000	0.0000	0.0000	0.0000	0.0000
*F*_IS_	−0.0270	−0.0320	−0.0590	—	—	—	—	—
HW	1.0000	1.0000	1.0000	—	—	—	—	—

*N*: number of sample; Ar: allele richness; *H*_*O*_: observed heterozygosity; *H*_*E*_: expected heterozygosity; *F*_IS_: inbreeding coefficient; *P < 0.05, indicative adjusted nominal level (5%): 0.00042.

**Table 2:** Hierarchical analysis of molecular variance (AMOVA) in *T. tambroides*.

Source of variation	Sum of squares	Variance components	Percentage of variation
Among populations	101.1190	0.3578	13.1257*
Among individuals within populations	352.2580	0.0800	2.9354
Within individuals	347.5000	2.2882	83.9389*

**Table 3:** Estimates of pairwise genetic distances ([11] below diagonal) and *F*_ST_ ([12] upper diagonal) among eight populations of *T. tambroides*.

	Negeri Sembilan	Pahang	Perak	Kelantan	Endau-Rompin	Batang Ai	Baleh	Ulu Limbang
(1) Negeri Sembilan	—	0.0166	0.0406*	0.3706*	0.1004*	0.0952*	0.0604*	0.0658*
(2) Pahang	0.0205	—	0.0103	0.3953*	0.1042*	0.0656*	0.0438*	0.0499*
(3) Perak	0.0390	0.0187	—	0.4019*	0.1249*	0.1251*	0.0967*	0.1043*
(4) Kelantan	0.1276	0.1418	0.1709	—	0.1811*	0.6494*	0.4753*	0.5717*
(5) Endau-Rompin	0.0549	0.0574	0.0721	0.0475	—	0.0724*	0.0011	0.0203*
(6) Batang Ai	0.0831	0.0668	0.1245	0.1125	0.0438	—	0.0057	0.0044
(7) Baleh	0.0569	0.0494	0.0954	0.0413	0.0116	0.0240	—	0.0444
(8) Ulu Limbang	0.0602	0.0512	0.0980	0.0655	0.0199	0.0028	0.0220	—

*P < 0.05.

**Table 4:** Results of assignment tests based on microsatellite gene frequencies of *T. tambroides*.

Assignment location										
	Negeri Sembilan	Pahang	Perak	Kelantan	Endau-Rompin	Baleh	Ulu Limbang	Batang Ai	*N*	% CA*
*Origin location *										
Negeri Sembilan	8	7	5						20	40.0
Pahang		9	8						17	52.9
Perak		5	14						19	72.6
Kelantan				0	20				20	0.00
Endau-Rompin	2	10	11		33	1	1	3	61	54.0
Baleh		3			2	0			5	0.00
Ulu Limbang		3				1	0	1	5	0.00
Batang Ai	1	1					1	2	5	40.0

Total									152	42.8

*CA: correct assignment.

**Table 5:** *P* values from analyses designed to identify evidence for recent bottlenecks* within eight populations of *T. tambroides*.

	IAM	TPM			SMM	Mode shift?
		60	70	80		
(1) Negeri Sembilan	0.1231	0.5771	0.6377	0.6377	0.8984	Y
(2) Pahang	0.0771	0.2661	0.3013	0.3394	0.5185	N
(3) Perak	0.0034*	0.0342*	0.0342*	0.0342*	0.2334	Y
(4) Kelantan	0.5000	0.5000	0.5000	0.5000	0.5000	N
(5) Endau-Rompin	0.5417	0.0398*	0.0085*	0.0040*	0.0012*	N
(6) Batang Ai	0.1748	0.0537*	0.0537*	0.0210*	0.0161*	Y
(7) Baleh	0.4131	0.0830	0.0830	0.0830	0.6250	Y
(8) Ulu Limbang	0.4143	0.0803	0.0398*	0.0266*	0.0215*	N

^#^IAM: infinite-alleles model; TPM: two-phase model; SMM: stepwise-mutational model. Under results from TPM analyses, values reflect the percent of mutations that adhered to a strict stepwise mutational model. Y: yes; N: no.

*Significant results (*P* < 0.05).
